# Acetylene-Fueled Trichloroethene Reductive Dechlorination in a Groundwater Enrichment Culture

**DOI:** 10.1128/mBio.02724-20

**Published:** 2021-02-02

**Authors:** Sara Gushgari-Doyle, Ronald S. Oremland, Ray Keren, Shaun M. Baesman, Denise M. Akob, Jillian F. Banfield, Lisa Alvarez-Cohen

**Affiliations:** a Department of Civil and Environmental Engineering, University of California, Berkeley, California, USA; b U. S. Geological Survey, Menlo Park, California, USA; c Department of Earth and Planetary Sciences, University of California, Berkeley, California, USA; d U. S. Geological Survey, Reston, Virginia, USA; e Earth and Environmental Sciences Division, Lawrence Berkeley National Laboratory, Berkeley, California, USA; The Hebrew University of Jerusalem

**Keywords:** acetylene, TCE, dechlorination, enrichment, trichloroethene

## Abstract

In aquifers, acetylene (C_2_H_2_) is a product of abiotic degradation of trichloroethene (TCE) catalyzed by *in situ* minerals. C_2_H_2_ can, in turn, inhibit multiple microbial processes including TCE dechlorination and metabolisms that commonly support dechlorination, in addition to supporting the growth of acetylenotrophic microorganisms. Previously, C_2_H_2_ was shown to support TCE reductive dechlorination in synthetic, laboratory-constructed cocultures containing the acetylenotroph *Pelobacter* sp. strain SFB93 and Dehalococcoides mccartyi strain 195 or strain BAV1. In this study, we demonstrate TCE and perchloroethene (PCE) reductive dechlorination by a microbial community enriched from contaminated groundwater and amended with C_2_H_2_ as the sole electron donor and organic carbon source. The metagenome of the stable, enriched community was analyzed to elucidate putative community functions. A novel anaerobic acetylenotroph in the phylum *Actinobacteria* was identified using metagenomic analysis. These results demonstrate that the coupling of acetylenotrophy and reductive dechlorination can occur in the environment with native bacteria and broaden our understanding of biotransformation at contaminated sites containing both TCE and C_2_H_2_.

## Abstract (precis)

Understanding the complex metabolisms of microbial communities in contaminated groundwaters is a challenge. PCE and TCE are among the most common groundwater contaminants in the United States that, when exposed to certain minerals, exhibit a unique abiotic degradation pathway in which C_2_H_2_ is a product.

## INTRODUCTION

Acetylene (C_2_H_2_) is a colorless, volatile hydrocarbon that is present in trace amounts in Earth’s modern atmosphere (1). In microbiology, C_2_H_2_ is commonly known as an inhibitor of microbial processes (2), including trichloroethene (TCE) reductive dechlorination (3) and supporting metabolisms such as fermentation (4), nitrogen fixation (5), denitrification (6), and methane oxidation (7). Despite its role as an inhibitor, C_2_H_2_ can also be used as a metabolic substrate supporting the growth of anaerobic and aerobic acetylenotrophic microorganisms (8). To date, there are 15 known strains of acetylenotrophs; aerobic acetylenotrophs include Mycobacterium lacticola, multiple *Rhodococcus* spp., and *Bacillus* spp. (8). To date, the only known anaerobic acetylenotrophs belong to the genus *Pelobacter* including P. acetylenicus and *Pelobacter* sp. strain SFB93 (8–10). These *Pelobacter* strains use acetylene hydratase to hydrate acetylene which is then fermented to produce H_2_, acetate, and ethanol (9–12). The enzyme from *P. acetylenicus* is the only well-characterized acetylene hydratase and is suggested to be structurally different from acetylene-transforming enzymes found in aerobic acetylenotrophs, although no such enzyme from aerobic acetylenotrophs has yet been characterized (13).

H_2_ is widely utilized as an electron donor for many microbial metabolisms, including TCE and perchloroethene (PCE) reductive dechlorination (14, 15). TCE and PCE are toxic chlorinated solvents that have become prevalent groundwater contaminants due to improper storage and handling practices (16). The only organisms known to reductively dechlorinate PCE and TCE entirely to the benign end product ethene are bacterial Dehalococcoides mccartyi strains (14, 17–19). *D. mccartyi* has strict requirements for exogenous substances such as hydrogen as electron donor, acetate as carbon source, and cofactor vitamin B_12_ (14, 15, 20, 21). Even when all metabolic requirements are met, *D. mccartyi* commonly does not grow well in axenic culture (15, 20). Since *D. mccartyi* strains are heavily reliant on other members of their microbial communities for robust growth, previous studies have focused on the characterization of constructed consortia and environmental enrichments to develop sustainable PCE and TCE bioremediation strategies (3, 15, 21–28).

Interestingly, C_2_H_2_ can be produced at TCE-contaminated sites by means of the abiotic transformation of TCE catalyzed by zero-valent iron or other minerals including iron sulfide (29–32). A number of previous studies have evaluated the effects of acetylene and other inhibitors on TCE dechlorination by *D. mccartyi* strains (3, 28, 33, 34) and consumption of acetylene produced by abiotic TCE degradation (35). However, the promotion of PCE and TCE bioremediation driven by their abiotic transformation products has been much less studied. Mao et al. reported the successful utilization of acetylene as the sole electron donor supporting TCE dechlorination in laboratory-constructed cocultures containing the acetylenotroph *Pelobacter* sp. strain SFB93 with *D. mccartyi* strains (3). That study also reported successful utilization of acetylene as the sole electron donor in a TCE-dechlorinating enrichment culture bioaugmented with *Pelobacter* sp. strain SFB93 (3). Based on the results of the laboratory-based experiments, we hypothesize that this unique coupling of acetylenotrophy and TCE dechlorination occurs in natural systems. As C_2_H_2_ is known to inhibit TCE dechlorination and organisms supporting biotic TCE dechlorination (26, 36), it is important to develop engineered solutions for remediation of TCE-contaminated sites with conditions promoting acetylene production.

In this study, we cultivated a groundwater enrichment culture that utilized C_2_H_2_ as the sole electron donor and organic carbon source while reducing PCE and TCE to vinyl chloride (VC). The culture (36BR-A-TCE) was enriched from groundwater collected from well 36BR-A at the Naval Air Warfare Center (NAWC) in Trenton, NJ. 16S rRNA gene analysis was conducted to identify the community structure within the microcosms, and metagenomic analysis was performed to determine the organisms responsible for acetylenotrophy. A novel anaerobic acetylenotroph was identified from the phylum *Actinobacteria*, which contains no other reported anaerobic acetylenotrophs. The results of this study will assist in the development of robust bioremediation strategies at complex contaminated sites.

## RESULTS

### C_2_H_2_ fuels PCE and TCE dechlorination in 36BR-A-TCE.

Anaerobic, acetylenotrophic enrichment cultures were established from groundwater collected from NAWC wells 36BR-A and 73BR-D2 with the addition of mineral medium to provide nutrients and C_2_H_2_ as the sole electron donor and organic carbon source (see Table S1 and Fig. S1 in the supplemental material). Groundwater amended with acetylene (but no nutrients) showed no consumption of acetylene (Fig. S1). Cultures established from well 36BR-A consumed the most acetylene (50 to 437 μmoles) and were the only cultures that produced methane over the course of incubation (Fig. S1). Therefore, the NAWC acetylenotrophic culture 36BR-A1 was selected for subsequent enrichment with chlorinated solvents.

10.1128/mBio.02724-20.1 FIG S1 Acetylene uptake (closed symbols) and methane production (red, open symbols) by Naval Air Warfare Center (NAWC) groundwaters from wells 36BR-A (A, B, and C) and 73BR-D2 (D, E, and F). Acetylenotrophic communities were enriched under anaerobic conditions with the addition of SeFr1 mineral medium in a 1:1 ratio with groundwater to provide nutrients and acetylene as the sole carbon and energy source. Panels C and F were full-strength well water without the addition of medium. Note that panels A and B and panels D and E are replicates started at different initial acetylene concentrations. Concentrations given in total micromoles per bottle (dissolved plus headspace). Download FIG S1, TIF file, 2.6 MB. Copyright © 2021 Gushgari-Doyle et al. 2021 Gushgari-Doyle et al. This content is distributed under the terms of the Creative Commons Attribution 4.0 International license.

10.1128/mBio.02724-20.4 TABLE S1 List of acetylenotrophic enrichment cultures established from NAWC groundwater samples collected on 11 June 2015. Download Table S1, DOCX file, 0.01 MB. Copyright © 2021 Gushgari-Doyle et al. 2021 Gushgari-Doyle et al. This content is distributed under the terms of the Creative Commons Attribution 4.0 International license.

**FIG 1:**
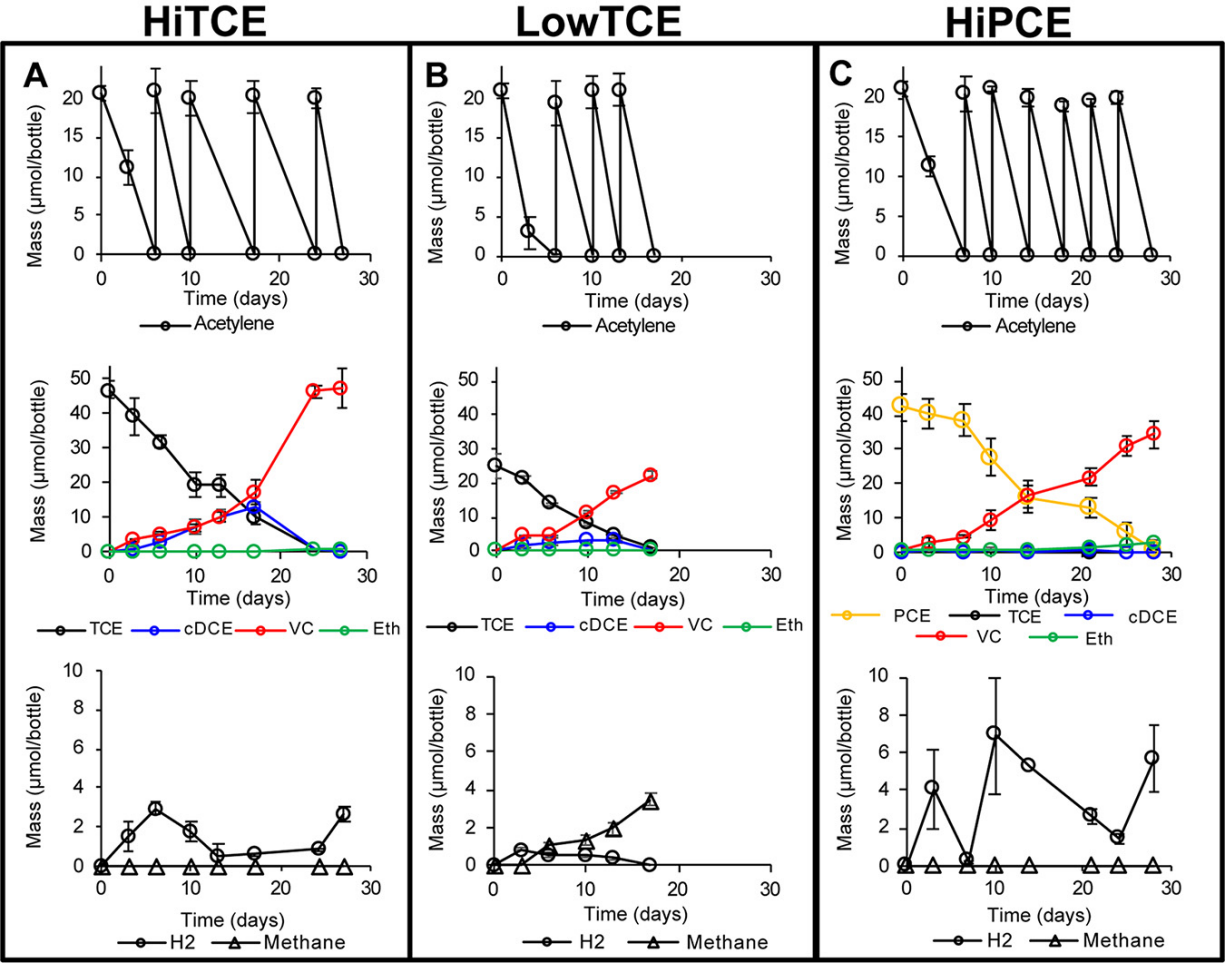
C_2_H_2_ consumption (top row), ethene and chlorinated ethene consumption and production (middle row), and H_2_ and methane production (bottom row) for HiTCE (A), LowTCE (B), and HiPCE (C) growth conditions. Quantities shown in total mass (dissolved plus headspace) per 160-ml serum bottle (100-ml liquid volume). Each acetylene peak denotes external amendment of acetylene. Error bars represent 1 standard deviation of experimental triplicates.

Culture 36BR-A-TCE was enriched from the NAWC acetylenotrophic culture 36BR-A1 using C_2_H_2_ as the sole electron donor and organic carbon source, TCE as the terminal electron acceptor, and CO_2_ as inorganic carbon source. Complete fermentation of 1 mol C_2_H_2_ produces 1 mol hydrogen (H_2_), which can then serve as the electron donor to reduce 0.5 mol TCE to VC (3). 36BR-A-TCE cultures were amended with 50 μmol TCE (HiTCE), 25 μmol TCE (LowTCE), or 50 μmol PCE (HiPCE) per bottle. C_2_H_2_ was readily consumed within 7 days of each amendment under all conditions, and 100 ± 9.4 μmol, 82 ± 7.5 μmol, and 140 ± 6.6 μmol C_2_H_2_ per bottle was amended over the course of the experiment in HiTCE (Fig. 1A), LowTCE (Fig. 1B), and HiPCE (Fig. 1C), respectively. Under the HiTCE condition, 47 ± 2.4 μmol of TCE was reduced and 47 ± 5.7 μmol of VC per bottle accumulated as the primary reduction product, with trivial amounts of *cis*-dichloroethene (cDCE) and ethene accumulated (Fig. 1A). Aqueous H_2_ concentrations in HiTCE remained below 2.9 μmol/bottle during TCE dechlorination (Fig. 1A), indicating that the H_2_ generation rate was roughly equal to its consumption rate and that interspecies hydrogen transfer occurred in the enrichment. No methane production was observed in HiTCE. Under LowTCE conditions, 24 ± 3.3 μmol of TCE was reduced and 22 ± 0.9 μmol of VC accumulated per bottle, with trivial amounts of *cis*-DCE and ethene (Fig. 1B). Aqueous H_2_ concentrations in LowTCE ranged from 0.04 to 0.88 μmol/bottle during TCE dechlorination, and 3.5 ± 0.3 μmol/bottle methane was produced over the course of the experiment (Fig. 1B). Under HiPCE conditions, 42 ± 4.1 μmol of PCE was reduced and 41 ± 3.5 μmol of VC and 6.1 ± 1.3 μmol ethene accumulated per bottle with trivial amounts of TCE and *cis*-DCE (Fig. 1C). Aqueous H_2_ concentrations in HiPCE ranged from 0.25 to 6.9 μmol/bottle during PCE-dechlorination, and no methane was produced over the course of the experiment (Fig. 1C). Assuming complete fermentation of C_2_H_2_, all H_2_ produced by C_2_H_2_ fermentation under all 3 growth conditions (HiTCE, LowTCE, and HiPCE) could be accounted for by chlorinated solvent reduction, methane production, and residual H_2_ measurement within 5% error.

### Composition of acetylenotrophic enrichment cultures.

16S rRNA gene analysis was initially performed to identify community structure in 36BR-A-TCE, the NAWC acetylenotrophic enrichment cultures, and the commercial dechlorinating community KB-1, which was augmented to the NAWC field site in 2008 (37, 38). It was determined that no known anaerobic acetylenotrophs, e.g., Pelobacter acetylenicus and *Pelobacter* sp. SFB93 from the family *Desulfuromonadaceae* (8), were present in any of the cultures (Fig. S1). An in-depth description of all samples and community diversity can be found in Tables S1, S2, and S3.

10.1128/mBio.02724-20.5 TABLE S2 Diversity indices for communities enriched from NAWC groundwaters on acetylene (and TCE, when applicable). Operational taxonomic units (OTUs) were based on a 97% sequence similarity cutoff. Inverse Simpson diversity index (InvS) with lower (lci) and higher (hci) 95% confidence intervals are reported. N/A, not applicable. Download Table S2, DOCX file, 0.01 MB. Copyright © 2021 Gushgari-Doyle et al. 2021 Gushgari-Doyle et al. This content is distributed under the terms of the Creative Commons Attribution 4.0 International license.

10.1128/mBio.02724-20.6 TABLE S3 Family-level affiliation and relative abundance (%) of OTUs representing >2% total relative abundance in NAWC acetylenotrophic enrichment cultures. OTUs were based on a 97% sequence similarity cutoff. n.d. indicates taxa that were not detected, and <2 indicates taxa that were less than 2% of the total reads in that sample. Download Table S3, DOCX file, 0.02 MB. Copyright © 2021 Gushgari-Doyle et al. 2021 Gushgari-Doyle et al. This content is distributed under the terms of the Creative Commons Attribution 4.0 International license.

**FIG 2:**
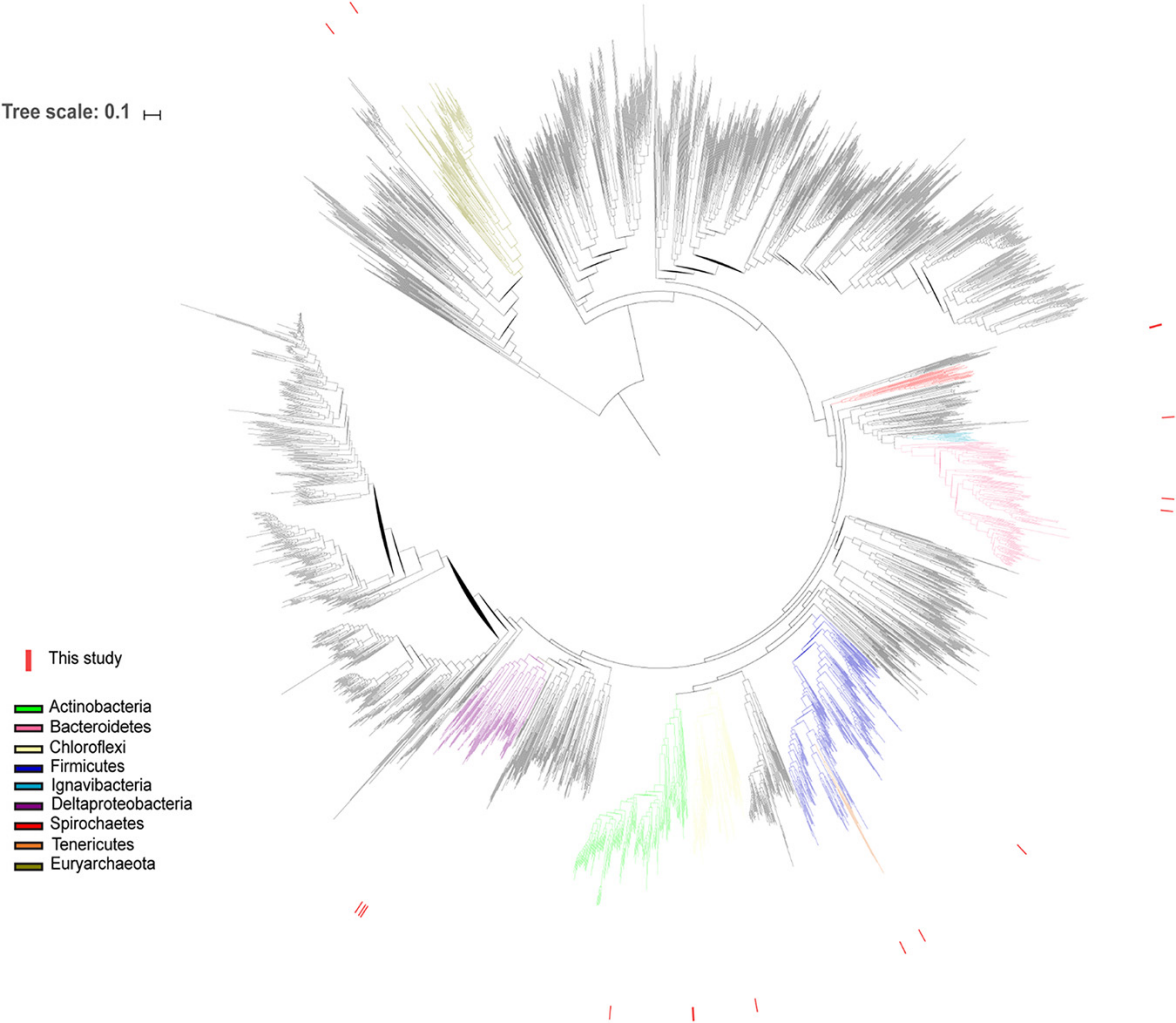
Maximum likelihood phylogenetic tree of organisms in the TCE/acetylene-consuming enrichment culture 36BR-A-TCE and reference organisms from *Bacteria* and *Archaea*. Phyla found in the enrichment are colored, and red marks around the perimeter identify organisms from this study.

To identify the acetylenotroph in 36BR-A-TCE, a genome-centric metagenomic analysis was performed on 36BR-A-TCE, resulting in 10 nearly complete (>90% complete) genomes, three genomes of good quality (>75% complete), and five moderately complete (55 to 65% complete) genomes (Fig. 2 and Table S4). From the metagenome sequence reads, the recovered *D. mccartyi* genome, presumed to be responsible for TCE dechlorination in 36BR-A-TCE, exhibited a relative community abundance of 40.6%. The genome for *D. mccartyi* encoded 7 reductive dehalogenase proteins *rdhA* and their corresponding anchor proteins *rdhB*. The average nucleotide identity (ANI) of *D. mccartyi* from 36BR-A-TCE was compared with 32 *D. mccartyi* strains with complete genomes available from the NCBI database (Fig. S2) and exhibited highest similarity (>99.5%) to *D. mccartyi* strain DCMB5. The qPCR analysis of reductive dehalogenase genes showed that *tceA* was present in (460 ± 100) × 10^6^ copies/ml, *bvcA* was present in (1.2 ± 0.1) × 10^6^ copies/ml, and *vcrA* was not observed in the enrichment.

10.1128/mBio.02724-20.2 FIG S2 Average nucleotide identity comparison among *D. mccartyi* strains from NAWC (***) and 32 known *D. mccartyi* strains from the Pinellas (left panel), Victoria (right panel, top), and Cornell (right panel, bottom) clades. Vertical dotted line designates a 95% average nucleotide identity. Download FIG S2, TIF file, 2.7 MB. Copyright © 2021 Gushgari-Doyle et al. 2021 Gushgari-Doyle et al. This content is distributed under the terms of the Creative Commons Attribution 4.0 International license.

10.1128/mBio.02724-20.7 TABLE S4 Genome features table from genome-resolved metagenomic analysis. The taxonomy column provides the most specific taxonomic level to which the genome can be classified and lists the level in parentheses. In data available in ggKbase, all bin names are preceded with “LAC_acetylene_”. Download Table S4, DOCX file, 0.02 MB. Copyright © 2021 Gushgari-Doyle et al. 2021 Gushgari-Doyle et al. This content is distributed under the terms of the Creative Commons Attribution 4.0 International license.

**FIG 3:**
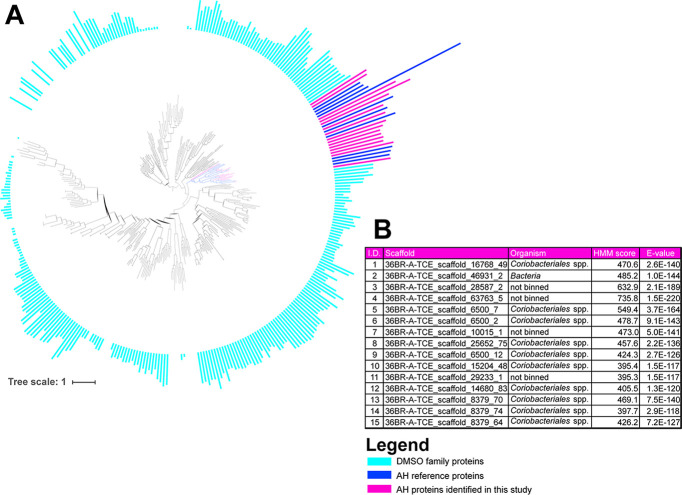
(A) Maximum likelihood tree of MopB superfamily of proteins. Branches colored dark blue are proteins that fall within the acetylene hydratase (AH) cluster from published references. Branches colored magenta are identified AH proteins from this study. Bars around the perimeter quantify the HMM score corresponding to each protein. Magenta bars are identified starting with 1 at the top to 15 at the bottom. DMSO, dimethyl sulfoxide. (B) The table details the scaffold containing the protein-encoding gene, binned organism, and HMM score of the protein. All *Coriobacteriales* scaffolds are from a single binned genome. The HMM score cutoff for AH identification in this analysis was 385.

**FIG 4:**
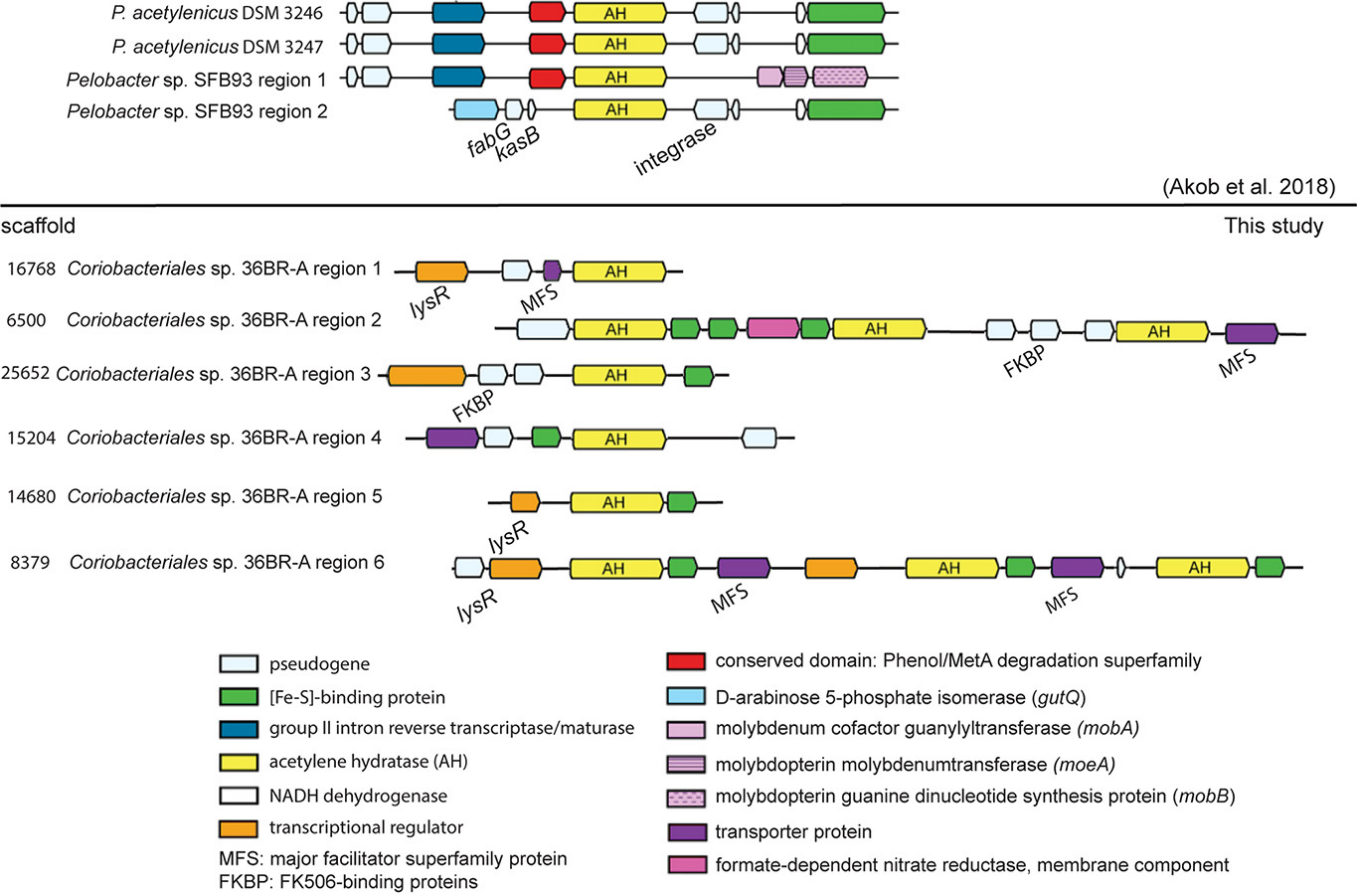
Genomic context of acetylene hydratase genes in *Coriobacteriales* spp. (this study) versus *P. acetylenicus* and *Pelobacter* sp. SFB93 (8).

The *Coriobacteriales* genome exhibited the second-highest relative abundance at 28.5% of the community. The hidden Markov model (HMM) constructed in this study for identification of putative acetylene hydratases yielded a significant similarity of the identified genes to known acetylene hydratases (scores = [395.3, 735.8], cutoff score = 385.0, E values = [1.0 × 10^−117^, 1.5 × 10^−220^], cutoff E value = 1.9 × 10^−113^), and the identified genes clustered within the acetylene hydratase cluster of the MopB superfamily (Fig. 3A and B). Based on the HMM analysis, the genes most likely to be responsible for acetylenotrophic metabolism in 36BR-A-TCE were from the *Coriobacteriales* genome. The genomic contexts of the identified genes were compared to those of the anaerobic acetylenotrophs *P. acetylenicus* and *Pelobacter* sp. SFB93 (Fig. 4). NCBI BLASTp was performed on amino acid sequences inferred from protein-encoding genes surrounding the acetylene hydratase genes in all three compared genomes, including the phenol/MetA degradation superfamily proteins, which were found to be transporters. Other metabolic genes found in the *Coriobacteriales* genome include those for nitrate and nitrite reduction (LAC_acetylene_scaffold_13414_67-68), sulfate and sulfite reduction (LAC_acetylene_scaffold_8379_87), and fermentation (multiple scaffolds), indicating this organism is a facultative acetylenotroph.

We identified two methanogenic archaea in 36BR-A-TCE: Methanosaeta concilii and *Methanobacterium* species with a combined 12 genes encoding subunits of methanogenesis marker gene methyl-coenzyme M reductase.

## DISCUSSION

In this study, we report the first successful environmental enrichment with the capacity to dechlorinate PCE and TCE to VC using acetylene as the sole electron donor and organic carbon source via fermentation. These results demonstrate that microbial communities in chlorinated solvent-contaminated groundwater have the potential for dechlorination supported by acetylenotrophy.

In 36BR-A-TCE, *D. mccartyi* was responsible for PCE and TCE dechlorination, while acetylenotrophy was attributed to *Coriobacteriales* spp. The enrichment also exhibited methanogenesis under LowTCE conditions, likely performed by Methanosaeta concilii and *Methanobacterium* spp., as indicated by the metagenomic analysis. In LowTCE, between 46 and 75% of produced H_2_ was used to reduce TCE to VC and between 13 and 20% was used to produce methane. C_2_H_2_ has been previously shown to inhibit both methanogenesis and reductive dehalogenation (3, 39), while high concentrations of TCE have been shown to inhibit methanogenesis (40). However, continuous C_2_H_2_ fermentation allowed for TCE dechlorination and methanogenesis to both occur under LowTCE conditions by removing the potential inhibitor and providing H_2_ and acetate to the enrichment, which are both metabolic requirements of *D. mccartyi* (14). In this study, H_2_ mass balances indicated that 1 mol C_2_H_2_ yielded 1 mol H_2_ and PCE/TCE reductive dechlorination and methanogenesis accounted for all H_2_ consumption within 5% error.

Microbial community characterization based on 16S rRNA gene sequence analysis showed similarity between communities in acetylenotrophic enrichment cultures from NAWC groundwater wells 36BR-A and 73BR-D2 (see Fig. S3 in the supplemental material). Similar phyla were reported using 16S rRNA gene sequencing and metagenomic analysis in 36BR-A-TCE. The presence of *Actinobacteria* in enrichments from both 36BR-A and 73BR-D2 groundwaters and lack thereof in the KB-1 commercial culture indicate that the acetylenotrophic *Coriobacteriales* species (*Actinobacteria*) was indigenous to the NAWC site. Additionally, the increase in relative abundance of *Dehalococcoidaceae* (the family to which belongs TCE-dechlorinating *D. mccartyi*) from 36BR-A1 to 36BR-A-TCE supports that *D. mccartyi* is responsible for PCE and TCE dechlorination. It should be noted that differences in relative abundance of community members in 36BR-A-TCE between 16S rRNA gene sequencing and metagenomic analysis may be due to copy number variance in the 16S rRNA gene or differences in sequencing depth.

10.1128/mBio.02724-20.3 FIG S3 Composition of microbial communities enriched on acetylene and/or TCE from NAWC groundwater wells 36BR-A and 73BR-D2. Also shown is the microbial community composition for KB-1, the dechlorinating enrichment culture used in the 2008 NAWC bioaugmentation experiment. Other Phyla represent organisms that make up less than 1% of total reads in all samples (*Acetothermia*, *Acidobacteria*, *Aminicenantes*, Archaea_unclassified, *Armatimonadetes*, *Caldiserica*, Candidate_division_OP3, Candidate_division_SR1, *Chlamydiae*, *Cloacimonetes*, *Cyanobacteria*, *Deferribacteres*, *Deinococcus*-*Thermus*, *Elusimicrobia*, *Fibrobacteres*, *Fusobacteria*, *Gemmatimonadetes*, GOUTA4, *Gracilibacteria*, *Hydrogenedentes*, *Latescibacteria*, *Lentisphaerae*, *Microgenomates*, Miscellaneous_Crenarchaeotic_Group, *Nitrospirae*, *Omnitrophica*, *Parcubacteria*, *Planctomycetes*, *Epsilonproteobacteria*, Proteobacteria_unclassified, *Synergistetes*, TA06, *Tenericutes*, *Thaumarchaeota*, *Thermotogae*, TM6, *Verrucomicrobia*, *Woesearchaeota* [DHVEG-6]). Download FIG S3, TIF file, 2.8 MB. Copyright © 2021 Gushgari-Doyle et al. 2021 Gushgari-Doyle et al. This content is distributed under the terms of the Creative Commons Attribution 4.0 International license.

The *D. mccartyi* strain in culture 36BR-A-TCE contains the reductive dehalogenase *tceA*, as evident by qPCR with gene-specific probes and primers and confirmed with metagenome analysis. Reductive dehalogenase *bvcA* was also found in 36BR-A-TCE via qPCR; however, it was present at concentrations 2 orders of magnitude lower than *tceA.* To investigate the presence of *bvcA*, a protein-protein BLAST was performed using BvcA against all predicted proteins in the 36BR-A-TCE metagenome, which returned one positive hit of an unnamed reductive dehalogenase in the *D. mccartyi* genome (LAC_acetylene_scaffold_8379_87, E value = 9e−131). Due to sequence similarity (but not full sequence identity), it is likely that the *bvcA* primers amplified this reductive dehalogenase with low fidelity, causing a false-positive qPCR result for *bvcA.* DCMB5, the strain with the highest ANI to the 36BR-A-TCE *D. mccartyi* strain, also contains the reductive dehalogenase *tceA*. The reductive dehalogenase *bvcA* was initially identified in *D. mccartyi* BAV1 and has been shown to metabolically transform DCE and VC to ethene (41). Because significant VC dechlorination was not observed in the cultures, it is not likely that the VC is the metabolic substrate for the unnamed reductive dehalogenase that caused the *bvcA* qPCR result. The reductive dehalogenase *vcrA* was not found in 36BR-A-TCE.

DCMB5, the *D. mccartyi* strain most closely related to the one in 36BR-A-TCE, was isolated from a contaminated site in Bitterfeld, Germany, that was not bioaugmented with the KB-1 commercial community (42, 43). An ANI of >99.5% constitutes identification of the same strain (44). While the *D. mccartyi* strain from this study is in the same clade as the KB-1 strain, its phylogenetic proximity to DCMB5 rather than the KB-1 strains suggests this *D. mccartyi* strain was native to the NAWC site.

The *Coriobacteriales* species identified in this study is the first reported anaerobic acetylenotroph outside the Deltaproteobacteria phylum. Rather, *Coriobacteriales* belongs to the phylum *Actinobacteria*, the same phylum as aerobic acetylenotrophs *M. lacticola* and *Rhodococcus* spp. While organisms from the order *Coriobacteriales* have historically been associated with animal host microbiomes, there have been several reports of environmental *Coriobacteriales* in the past decade as more environmental communities are studied (45–50). The identified acetylene hydratase genes were analyzed using NCBI BLAST (51) against the genomes of *P. acetylenicus*, *Pelobacter* sp. SFB93, and aerobic acetylenotrophs belonging to the phylum *Actinobacteria*. It was found that the acetylene hydratases identified here show highest sequence similarity to acetylene hydratases and genes identified as molybdopterin oxidoreductase family proteins in *P. acetylenicus*, *Pelobacter* sp. SFB93, and Rhodococcus opacus (Table S5). The acetylene hydratase of *R. opacus* is known to differ from that of *P. acetylenicus* as it does not show cross-reactivity with antibodies raised to the enzyme of *P. acetylenicus* and it is molybdenum dependent, as opposed to tungsten dependent (12, 13). As *R. opacus* is an aerobic acetylenotroph, the results from NCBI BLAST indicate that enzymes using acetylene as the substrate have conserved regions, even between aerobic and anaerobic bacteria. It is important to note that the only acetylene hydratase enzyme isolated and characterized to date is that of *P. acetylenicus* (52, 53). Further research is required to biochemically verify the acetylene hydratases identified in this study and in other previous work.

10.1128/mBio.02724-20.8 TABLE S5 BLAST results (E value and percent [%] identity) of identified acetylene hydratase genes in culture 36BR-A-TCE against *P. acetylenicus, Pelobacter* sp. strain SFB93, and aerobic acetylenotrophs Rhodococcus rhodochrous, Rhodococcus opacus, Rhodococcus zopfii, and Gordonia alkanivorans. Download Table S5, DOCX file, 0.02 MB. Copyright © 2021 Gushgari-Doyle et al. 2021 Gushgari-Doyle et al. This content is distributed under the terms of the Creative Commons Attribution 4.0 International license.

Based on genomic context analysis and comparison of the acetylene hydratase genes, it can be deduced that Fe-S-binding proteins are important for acetylene hydratase function due to their presence nearly adjacent to all putative and reference acetylene hydratases but one (*Coriobacteriales* sp. scaffold 16768). In four of six *Coriobacteriales* scaffolds containing acetylene hydratases, transporter proteins (mostly from the major facilitator superfamily) are found adjacent. As the phenol/MetA degradation superfamily proteins found adjacent to *Pelobacter* acetylene hydratases are also transporter proteins, these transporters may also be essential to acetylene hydratase function. Lastly, the transcriptional regulator *lysR* was found in three of six acetylene hydratase-containing *Coriobacteriales* scaffolds, while the *marR* transcriptional regulator was found upstream of the *Pelobacter* sp. SFB93 acetylene hydratase (8, 54). The *lysR* and *marR* transcriptional regulators both contain helix-turn-helix motifs and have been found to regulate degradation of organic compounds, among many other cellular functions (55, 56). These similar genetic contexts suggest positive identification of the acetylene hydratase proteins in the *Coriobacteriales* species from 36BR-A-TCE.

The identification of an anaerobic acetylenotroph outside the Deltaproteobacteria phylum suggests that acetylenotrophy may be a more common metabolism than originally thought, as postulated by Akob et al. (8). Future research efforts aimed at isolating and characterizing the organism responsible for acetylenotrophy in this enrichment culture, as well as isolating and characterizing more acetylene hydratase enzymes, will increase knowledge of this relatively undescribed metabolism. Furthermore, it has become increasingly clear over the past few decades that organisms performing reductive dehalogenation, including *D. mccartyi* strains, are commonly found in a variety of environments. In this work, PCE and TCE were dechlorinated to VC. In future work, or for application of this community physiology for bioremediation, additional optimization should be enacted to increase ethene yield by the presence of VC reducers *in situ* or bioaugmentation with a *D. mccartyi* strain capable of metabolic transformation of VC to ethene. As characterization and remediation strategies for single environmental contaminants become more robust, it is important to begin synthesizing remediation strategies that cover a broader range of potential environments. This study begins to identify potential remediation strategies for environments that fostered production of acetylene in PCE- and TCE-contaminated sites.

## MATERIALS AND METHODS

### Chemicals.

TCE and PCE (99.6%, American Chemical Society [ACS] reagent) were obtained from Acros Organics (Geel, Belgium). C_2_H_2_ (>99.2%) was obtained from Praxair, Inc. (San Ramon, CA, USA), or generated in the laboratory by reacting calcium carbide (CaC_2_) with water. All other chemicals used were of reagent-grade quality or higher. All other gases (air, nitrogen, hydrogen, and nitrogen-CO_2_ mixture) were obtained from Praxair, Inc. (San Ramon, CA, USA).

### Field sampling.

The Naval Air Warfare Center (NAWC) site is a long-term U.S. Geological Survey (USGS) study site with extensive groundwater TCE contamination (57–59). The site is a 0.24-km^2^ facility in West Trenton, NJ, and the hydrogeology and extent of TCE, *cis*-dichloroethene (cDCE), and VC plumes have been previously described (57, 59). An *in situ* bioaugmentation experiment was initiated at the NAWC site on 15 October 2008 by injecting emulsified soybean oil and sodium lactate (EOS) as an electron donor and the KB-1 bacterial consortium as a source of dehalogenating bacteria (37, 38, 60). For this study, groundwater was collected from wells 36BR-A and 73BR-D2, which are injection and monitoring wells for the bioaugmentation experiment, respectively. Unfiltered groundwater samples were collected by peristaltic pumping into sterile, 1-liter amber glass bottles (without headspace) on 11 June 2015. Samples were stored on ice and shipped to the USGS in Menlo Park, CA. Upon arrival at the USGS, groundwater was transferred to crimp-sealed, nitrogen-flushed serum bottles and stored at 5°C until further analysis.

### Bacterial cultures and growth conditions.

To enrich acetylenotrophs, groundwater samples were mixed with an equal volume of anaerobic freshwater culture medium SeFr1 (10, 61). SeFr1 (pH 7.3) contains, per liter, 0.225 g K_2_HPO_4_, 0.225 g KH_2_PO_4_, 0.46 g NaCl, 0.225 g (NH_4_)_2_SO_4_, 0.117 g MgSO_4_·7H_2_O, 0.06 g CaCl_2_·2H_2_O, 3 mg Na_2_WO_4_·2H_2_O, 4.2 g NaHCO_3_, 1.0 ml SL10 trace element solution (90), and 1.0 ml vitamin solution (61). Twenty milliliters of the 1:1 groundwater-SeFr1 mixture was then transferred to gastight 60-ml serum bottles with 100% nitrogen headspace and 0.05 ml or 0.1 ml 100% acetylene. When acetylene concentrations were depleted below detection using gas chromatography with flame ionization detection (GC-FID), more acetylene was added. After depletion of multiple acetylene doses, a 5-ml subsample of the enrichment culture was transferred to 50 ml of new SeFr1 medium and amended with 0.1 ml 100% acetylene. Bottles were incubated at 28°C in the dark without agitation. All cultivation was performed using anaerobic, aseptic techniques.

To enrich for TCE dechlorination, enrichment cultures from well 36BR-A were first initiated for acetylenotrophy and then for TCE dechlorination once stable acetylene consumption was observed. 36BR-A-TCE was grown in the BAV1 defined mineral salts medium (previously described) in 160-ml serum bottles with 100 ml liquid volume and 80:20 N_2_/CO_2_ headspace (21), 46 ± 1.5 μmol TCE as terminal electron acceptor, 230 ± 11 μmol acetylene as sole electron donor and additional carbon source, and vitamins including 100 μM vitamin B_12_ (62). 36BR-A-TCE was grown under these conditions and continuously subcultured for 18 months to achieve a stable community structure before the described experimental characterization. Heat-killed controls were created by autoclaving (121°C, 203 kPa, 1 h) and monitored (data not shown). Bottles were incubated at 28°C without light or shaking.

### Analytical methods.

Acetylene concentrations in the initial acetylenotrophic enrichments were measured using GC-FID using a HayeSep A (80/100; 3.2 mm outside diameter [o.d.] × 2.44 m) column as described previously (10). Chloroethenes, ethene, methane, and acetylene were measured with 100-μl headspace samples on an Agilent 7890A gas chromatograph equipped with a flame ionization detector and a 30-m J&W capillary column with an inside diameter of 0.32 mm (Agilent Technologies, Santa Clara, CA, USA). A gradient temperature program method was used as previously described (63). Hydrogen was measured by reduced gas detection-gas chromatography using 100-μl headspace samples on a Peak Laboratories Peak Performer 1 gas chromatograph equipped with a reducing compound photometer (RCP) equipped with 16-inch Uni 1S 60/80 and 81-inch MS13X 60/80 columns according to the manufacturer’s instructions (Peak Laboratories, Mountain View, CA, USA). The mass of each compound was calculated based on gas-liquid equilibrium by using Henry’s law constants at 28°C according to mass (μmol/bottle) = *C*_l_ × *V*_l_ + *C_g_* × *V_g_*.

### DNA extraction and cell number quantification.

Liquid samples (1.5 ml) were collected for cell density measurements, and cells were pelleted by centrifugation (21,000 × *g*, 10 min at 4°C). Samples were taken from 36BR-A-TCE on day 27 of the HiTCE experiment, once all TCE was consumed. For all other enrichments, samples were taken after consumption of all amended acetylene (see Table S1 in the supplemental material). Genomic DNA of 36BR-A-TCE was extracted from cell pellets using the DNeasy blood and tissue kit (Qiagen, Valencia, CA, USA) according to the manufacturer’s instructions for Gram-positive bacteria. Genomic DNA of all other enrichments was extracted from cell pellets using the Mo Bio PowerSoil kit according to the manufacturer’s instructions (Mo Bio, CA). DNA quantification was performed using the Qubit 3 fluorometer (Invitrogen, Carlsbad, CA, USA) with the Qubit double-stranded DNA (dsDNA) high-sensitivity (HS) assay kit per manufacturer’s instructions. qPCR using SYBR green-based detection reagents was applied to quantify gene copy numbers of common reductive dehalogenases associated with TCE-reducing bacteria with *D. mccartyi tceA*, *bvcA*, and *vcrA* gene probes and primers as previously described (64).

### Microbial community structure analysis.

Liquid samples (20 ml) were collected for genomic DNA (gDNA) extraction and community structure analysis via 16S rRNA gene v4 hypervariable region. Cells were harvested and DNA extracted as described above. For 36BR-A-TCE, 10 μl of 10-ng/μl gDNA was sent to the QB3 Vincent J. Coates Genomics Sequencing Laboratory at UC Berkeley for Illumina 16S iTag sequencing. For all other enrichments, DNA extracts were sent to Michigan State University’s Research Technology Support Facility (RTSF; East Lansing, MI, USA) for Illumina 16S iTag sequencing. Microbial sequence data were processed for quality control, alignment, and taxonomic assignment using MOTHUR v.1.38.1, based on the Silva 123 nonredundant database (65–68) using the USGS Advanced Research Computing (ARC) Yeti high-performance computing facility. Diversity indices were calculated using MOTHUR v.1.38.1 (66).

### Metagenome community analysis.

A liquid sample (20 ml) was collected for gDNA extraction and community structure analysis via metagenome sequencing. Cells were harvested and DNA extracted as described above. Eighty microliters of 20-ng/μl gDNA was sent to the QB3 Vincent J. Coates Genomics Sequencing Laboratory at UC Berkeley for library preparation and sequencing on the Illumina HiSeq4000 (Illumina, San Diego, CA). The raw data sequence reads were processed and analyzed following the ggKbase standard operating procedure (SOP) (https://ggkbase-help.berkeley.edu/overview/data-preparation-metagenome/). In summary, Illumina adapters and trace contaminants were removed (BBTools, JGI), and raw sequences were quality-trimmed with Sickle (69). Paired-end reads were assembled using IDBA_UD with the precorrection option and default settings (70). For coverage calculations, reads were mapped with bowtie2 (71). Genes were predicted by Prodigal (72), and predicted protein sequences were annotated using usearch (73) against KEGG, UniRef100, and UniProt databases. The 16S rRNA gene and tRNA prediction was done with an in-house script and tRNAscan-SE (74), respectively. At this point, the processed data were uploaded to ggKbase for binning.

Manual binning was performed using the ggKbase tool. The parameters for binning were GC% and coverage (CV) distribution, phylogeny of the scaffolds, and single-copy gene inventory. Quality of the manual bins was assessed by the number of bacterial single-copy genes (BSCG) and ribosomal proteins (RP) found in each bin (aiming at finding the full set of genes, while minimizing the multiple copies). In addition to manual binning, automated binning was performed using four binners: ABAWACA1, ABAWACA2 (75), MetaBAT (76), and MaxBin2 (77). For all, the default parameters were chosen.

All bins from both automatic and manual binning tools were input into DASTool (78) to iterate through bins and choose the optimal set. CheckM was run to analyze genome completeness (79). The scaffold-to-bin file created by DASTool was uploaded back to ggKbase, and all scaffolds were rebinned to match the DASTool output. Each of the new bins was manually inspected and refined to remove scaffolds with aberrant GC, coverage, and phylogenetic profile. In all, 13.1 Gbp were sequenced and 98% of the reads were assembled into a total assembly length of 64.45 Mbp (minimum scaffold length of 1,000 bp). Of that, 39.67 Mbp were included in the refined bins.

Phylogenetic analysis of the genomes was based on a set of 15 ribosomal proteins that were selected because they are consistently colocated in a single genomic region, thus avoiding binning-based chimeras (80). Each gene was aligned separately to a set of 3,225 reference genomes, followed by concatenation while keeping the aligned length of each gene intact. A preliminary tree was created by adding the queried genomes to the reference tree using pplacer v1.1.alpha19 (81) and a set of in-house scripts. The tree was uploaded to iTOL (82) for visualization and editing. The final tree was constructed with RAxML in the CIPRES Science Gateway V. 3.3 (83). Relative abundance of organisms in the community was calculated based on (bin coverage)/(sum of all bin coverages).

Analysis of *D. mccartyi* strain diversity was determined by the dereplication tool, dRep (84), using a 99.5% threshold (strain level) for clustering.

### Hidden Markov model analysis.

A hidden Markov model (HMM) was developed for similarity analysis of putative acetylene hydratase proteins in the 36BR-A-TCE enrichment. The seed sequences used to create the HMM were the confirmed acetylene hydratases of *P. acetylenicus* and *Pelobacter* sp. strain SFB93 and additional sequences found using BLASTp in NCBI. Prior to creating the HMM, the sequence placement in the correct gene cluster within the MopB superfamily tree was checked. Reference sequences for the tree were taken from the NCBI Conserved Domain database as well as from the work of Castelle et al. (85). Sequences were aligned using MAFFT (86), and a tree was constructed with FastTree (87).

Once the seed sequences were selected, an HMM was created using the HMMER suite (88), and cutoff score was assigned based on scores from the reference sequences. The HMM was then used to search for homologous sequences in the metagenome using HMMER suite default parameters. The sequences above the cutoff were added to the reference sequences and aligned, and a RAxML tree was constructed. The tree with the HMM scores was visualized in iTOL.

### Data availability.

The metagenome of culture 36BR-A-TCE is available from the NCBI GenBank database under BioProject number PRJNA615907. 16S rRNA gene sequence data are deposited in the NCBI Sequence Read Archive under BioProject number PRJNA615907 and accession numbers SRX8026671 to SRX8026678. Resolved genomes from metagenome analysis have been made public (https://ggkbase.berkeley.edu/LAC_acetylene_final/organisms). Data from acetylenotrophic cultivation and dechlorination studies are available from the work of Baesman et al. (89).
