# Supplementary figures and images for: Acetylene-Fueled Trichloroethene Reductive Dechlorination in a Groundwater Enrichment Culture
Source: mBio. 2021 Feb 2;12(1):e02724-20. doi: 10.1128/mBio.02724-20 (PMC7858054; doi:10.1128/mBio.02724-20)

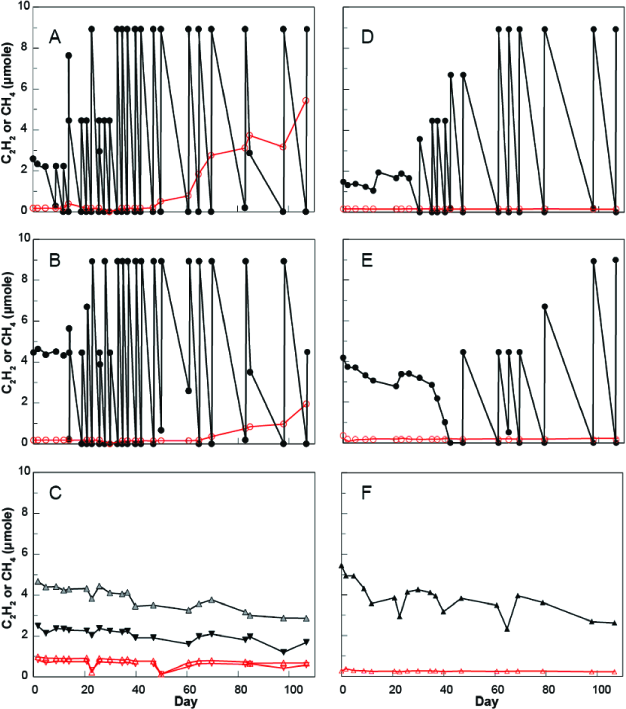

Supplement: FIG S1 [file mBio.02724-20-sf001.tif]

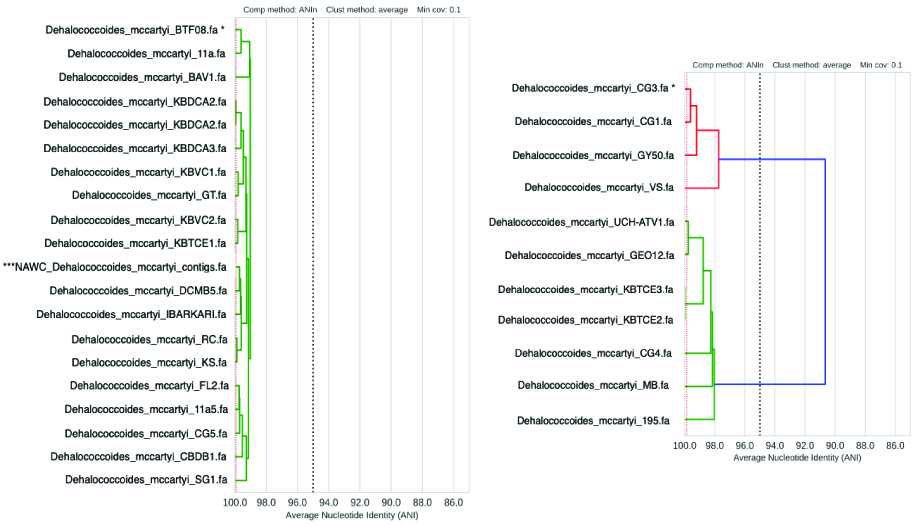

Supplement: FIG S2 [file mBio.02724-20-sf002.tif]

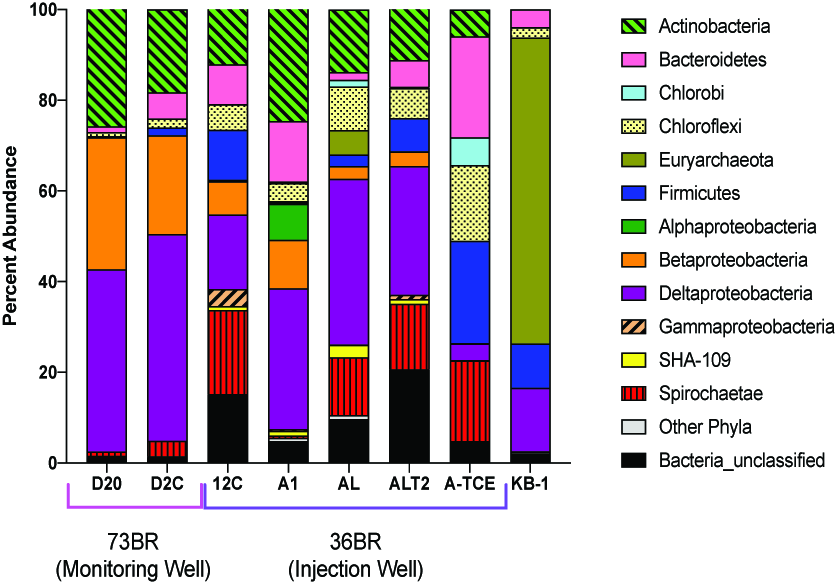

Supplement: FIG S3 [file mBio.02724-20-sf003.tif]
