# Supplementary material for: Acetylene-Fueled Trichloroethene Reductive Dechlorination in a Groundwater Enrichment Culture
Source: mBio. 2021 Feb 2;12(1):e02724-20. doi: 10.1128/mBio.02724-20 (PMC7858054; doi:10.1128/mBio.02724-20)
Supplement: TABLE S1 [file mBio.02724-20-st001.docx]

| **Groundwater Well** | **Culture Name** | **Date Initiated** | **Total µmoles Acetylene Added** | **Number of Acetylene Additions** |
| --- | --- | --- | --- | --- |
| 36BR-A | 36BR-AL | Transfer from culture 36BR-A-SB | 437.5 | 16 |
| 36BR-A | 36BR-ALT2 | Transfer from culture 36BR-AL | 187.5 | 9 |
| 36BR-A | 36BR-A-12C | 06 August 2015 | 290.2 | 11 |
| 36BR-A | 36BR-A1 | 06 April 2016 | 51.3 | 6 |
| 36BR-A | 36BR-A-TCE | Transfer from culture 36BR-A1; 05 October 2017 | 120.8* | 6* |
| 73BR-D2 | 73BR-DO | 06 April 2016 | 4.5 | 1 |
| 73BR-D2 | 73BR-D2C | 06 April 2016 | 4.5 | 1 |

*Under experimental conditions HiTCE
