# Supplementary material for: Acetylene-Fueled Trichloroethene Reductive Dechlorination in a Groundwater Enrichment Culture
Source: mBio. 2021 Feb 2;12(1):e02724-20. doi: 10.1128/mBio.02724-20 (PMC7858054; doi:10.1128/mBio.02724-20)
Supplement: TABLE S2 [file mBio.02724-20-st002.docx]

| **Groundwater Well** | **Culture Name** | **Library Name** | **No. Sequences** | **No. of OTUs** | **Coverage (%)** | **InvS** | **InvS_lci** | **InvS_hci** |
| --- | --- | --- | --- | --- | --- | --- | --- | --- |
| N/A | KB-1 | KB-1 | 32,906 | 280 | 0.995 | 2.13 | 2.10 | 2.16 |
| 36BR-A | 36BR-AL-t2 | ALT2 | 212,311 | 2863 | 0.988 | 9.98 | 9.93 | 10.0 |
|  | 36BR-A-12C | 12C | 216,664 | 2725 | 0.989 | 10.4 | 10.3 | 10.4 |
|  | 36BR-AL | AL | 221,781 | 3047 | 0.989 | 10.5 | 10.5 | 10.6 |
|  | 36BR-A1 | A1 | 206,460 | 2561 | 0.990 | 7.83 | 7.78 | 7.89 |
|  | 36BR-A-TCE | A-TCE | 137,002 | 211 | 0.999 | 7.17 | 7.13 | 7.21 |
| 73BR-D2 | 73BR-DO | D20 | 176,479 | 2780 | 0.986 | 5.33 | 5.31 | 5.35 |
|  | 73BR-D2C | D2C | 186,198 | 2537 | 0.988 | 7.46 | 7.43 | 7.49 |
