# Supplementary material for: Acetylene-Fueled Trichloroethene Reductive Dechlorination in a Groundwater Enrichment Culture
Source: mBio. 2021 Feb 2;12(1):e02724-20. doi: 10.1128/mBio.02724-20 (PMC7858054; doi:10.1128/mBio.02724-20)
Supplement: TABLE S3 [file mBio.02724-20-st003.docx]

| **Well** | | | **36BR-A** | | | | | **73BR-D2** | |  |
| --- | --- | --- | --- | --- | --- | --- | --- | --- | --- | --- |
| **Culture** | | | **12C** | **TCE** | **A1** | **AL** | **ALT2** | **D20** | **D2C** | **KB-1** |
| **Taxonomic Group (Phylum, Family)** | *Spirochaetae* | *Spirochaetaceae* | 18.5 | 17.7 | <2 | 12.6 | 14.4 | <2 | 3.4 | <2 |
|  | *SHA-109* | SHA-109_unclassified | <2 | <2 | <2 | 2.74 | <2 | n.d. | n.d. | n.d. |
|  | *Gammaproteobacteria* | *Pseudomonadaceae* | 3.71 | n.d. | <2 | n.d. | <2 | n.d. | n.d. | n.d. |
|  | *Deltaproteobacteria* | *Desulfomicrobiaceae* | n.d. | <2 | 22.4 | <2 | n.d. | <2 | <2 | n.d. |
|  |  | *Desulfovibrionaceae* | 10.5 | 2.45 | 7.82 | 7.69 | 8.37 | 17.5 | 30 | <2 |
|  |  | *Desulfuromonadales,* unclassified | <2 | <2 | <2 | 26.3 | 7.41 | 20.8 | 12 | 6.92 |
|  |  | *Geobacteraceae* | <2 | n.d. | n.d. | <2 | <2 | <2 | <2 | 6.30 |
|  |  | *Myxococcales*, unclassified | n.d. | n.d. | <2 | <2 | n.d. | <2 | 2.68 | n.d. |
|  |  | *Syntrophaceae* | 3.98 | <2 | <2 | <2 | 11.9 | <2 | <2 | <2 |
|  | *Betaproteobacteria* | *Comamonadaceae* | n.d. | n.d. | 4.67 | n.d. | n.d. | 2.61 | n.d. | n.d. |
|  |  | *Rhodocyclaceae* | 6.98 | n.d. | 5.55 | 2.21 | 3.12 | 25.9 | 19.6 | n.d. |
|  | *Alphaproteobacteria* | *Sphingomonadaceae* | <2 | <2 | 7.89 | <2 | <2 | <2 | <2 | n.d. |
|  | *Firmicutes* | *Erysipelotrichaceae* | <2 | 18.1 | <2 | <2 | <2 | n.d. | n.d. | n.d. |
|  |  | *Eubacteriaceae* | 10.9 | <2 | <2 | <2 | 7.17 | <2 | <2 | 9.34 |
|  | *Euryarchaeota* | *Methanosaetaceae* | n.d. | <2 | <2 | 2.76 | <2 | n.d. | n.d. | <2 |
|  |  | *Thermoplasmatales Incertae Sedis* | n.d. | n.d. | n.d. | <2 | n.d. | n.d. | n.d. | 67.5 |
|  | *Chloroflexi* | *Anaerolineaceae* | 5.51 | <2 | 3.41 | 7.71 | 6.56 | <2 | <2 | <2 |
|  |  | *Dehalococcoidaceae* | n.d. | 14.9 | <2 | <2 | n.d. | <2 | <2 | <2 |
|  | *Chlorobi* | SJA-28 | n.d. | 6.08 | <2 | <2 | n.d. | n.d. | n.d. | <2 |
|  | *Bacteroidetes* | B01R012 | <2 | n.d. | 2.39 | n.d. | n.d. | <2 | n.d. | n.d. |
|  |  | *Porphyromonadaceae* | 4.18 | n.d. | <2 | <2 | 2.71 | <2 | <2 | <2 |
|  |  | *Rikenellaceae* | 4.44 | 21 | 1.8 | <2 | 2.02 | <2 | 4.7 | <2 |
|  |  | WCHB1-69 | <2 | <2 | 8.6 | <2 | <2 | <2 | <2 | 3.55 |
|  | *Actinobacteria* | *Actinobacteria,* unclassified^1^ | 12.1 | 5.83 | 24.5 | 13.6 | 11.1 | <2 | <2 | n.d. |
|  |  | *Coriobacteriaceae* | <2 | <2 | <2 | <2 | <2 | 25.5 | 18.2 | n.d. |

^1^The sequence of the most abundant representative OTU for unclassified *Actinobacteria* was compared to the GenBank database via a BLAST search, which indicated this OTU is affiliated with the family *Coriobacteriaceae*.
