# Supplementary material for: Acetylene-Fueled Trichloroethene Reductive Dechlorination in a Groundwater Enrichment Culture
Source: mBio. 2021 Feb 2;12(1):e02724-20. doi: 10.1128/mBio.02724-20 (PMC7858054; doi:10.1128/mBio.02724-20)
Supplement: TABLE S4 [file mBio.02724-20-st004.docx]

| **bin name** | **taxonomy** | **bin length (bp)** | **GC%** | **coverage** | **relative abundance (%)** | **# contigs** | **# features** | **longest contig (bp)** | **completeness (%)** | **contamination (%)** |
| --- | --- | --- | --- | --- | --- | --- | --- | --- | --- | --- |
| Acetobacterium_woodii_46_9 | Acetobacterium woodii (species) | 1627966 | 46.3 | 9.0 | 0.2 | 836 | 1722 | 7415 | 56.8 | 0.6 |
| Bacteria_53_217 | Bacteria (domain) | 2437437 | 52.5 | 216.7 | 3.9 | 97 | 2287 | 99087 | 95.4 | 0.0 |
| Bacteria_61_14 | Bacteria (domain) | 2689526 | 60.7 | 14.5 | 0.3 | 403 | 2617 | 33416 | 88.6 | 2.3 |
| Actinomycetales_67_18_67_465 | Actinomycetales (order) | 3072537 | 67.1 | 465.2 | 8.4 | 36 | 2869 | 276997 | 94.7 | 1.0 |
| Bacteroidetes_43_11_43_111 | Bacteroidetes (phylum) | 2256804 | 43.0 | 111.3 | 2.0 | 57 | 1993 | 275347 | 96.2 | 1.2 |
| Bacteroidetes_37_22_48_12 | Bacteroidetes (phylum) | 2797339 | 47.7 | 12.5 | 0.2 | 626 | 2485 | 25751 | 90.7 | 0.7 |
| Coriobacteriales_65_1583 | Coriobacteriales (order) | 2739826 | 65.0 | 1583.0 | 28.5 | 89 | 2667 | 242507 | 97.1 | 5.8 |
| Coriobacteriales_66_28 | Coriobacteriales (order) | 2691303 | 65.6 | 28.1 | 0.5 | 126 | 2652 | 155867 | 96.3 | 2.6 |
| Dehalococcoides_mccartyi_47_2252 | Dehalococcoides mccartyi (species) | 1380472 | 47.4 | 2251.6 | 40.6 | 5 | 1494 | 574557 | 99.0 | 0.0 |
| Desulfovibrio_65_116 | Desulfovibrio (genus) | 2505780 | 65.2 | 116.1 | 2.1 | 52 | 2294 | 272761 | 75.2 | 0.0 |
| Desulfovibrio_69_12 | Desulfovibrio (genus | 1622666 | 69.0 | 11.8 | 0.2 | 528 | 1843 | 12136 | 63.8 | 2.4 |
| Firmicutes_46_580 | Firmicutes (phylum) | 2134531 | 45.8 | 580.2 | 10.5 | 3 | 2184 | 962161 | 98.1 | 1.1 |
| Ignavibacteriales_40_37 | Ignavibacteriales (order) | 1553393 | 40.1 | 36.7 | 0.7 | 307 | 1370 | 26900 | 55.5 | 0.6 |
| Methanobacterium_44_11 | Methanobacterium (genus) | 1229534 | 43.9 | 11.4 | 0.2 | 618 | 1236 | 10668 | 60.8 | 0.1 |
| Methanosaeta_concilii_53_16 | Methanosaeta concilii (species) | 2015823 | 53.3 | 15.6 | 0.3 | 230 | 2115 | 42110 | 93.5 | 1.3 |
| Mollicutes_58_46 | Mollicutes (class) | 1604845 | 58.1 | 45.7 | 0.8 | 48 | 1541 | 264917 | 93.3 | 0.0 |
| Syntrophobacter_fumaroxidans_62_10 | Syntrophobacter fumaroxidans (species) | 3317538 | 62.0 | 9.8 | 0.2 | 888 | 3179 | 20580 | 87.4 | 6.6 |
| Thermosinus_carboxydivorans_51_32 | Thermosinus carboxydivorans (species) | 1385754 | 51.4 | 32.2 | 0.6 | 462 | 1618 | 17587 | 57.1 | 12.9 |
