# Supplementary material for: Acetylene-Fueled Trichloroethene Reductive Dechlorination in a Groundwater Enrichment Culture
Source: mBio. 2021 Feb 2;12(1):e02724-20. doi: 10.1128/mBio.02724-20 (PMC7858054; doi:10.1128/mBio.02724-20)
Supplement: TABLE S5 [file mBio.02724-20-st005.docx]

­­

| LAC_acetylene_ | | *Pelobacter acetylenicus* | | *Pelobacter SFB93* | | *Rhodococcus rhodochrous* | | *Rhodococcus opacus* | | *Rhodococcus zopfii* | | *Gordonia alkanivorans* | |
| --- | --- | --- | --- | --- | --- | --- | --- | --- | --- | --- | --- | --- | --- |
|  |  | e-value | % identity | e-value | % identity | e-value | % identity | e-value | % identity | e-value | % identity | e-value | % identity |
| 1 | scaffold_16768_49 | 2.0E-90 | 31% | 2.0E-86 | 30% | 6.0E-50 | 26% | 1.0E-109 | 33% | 1.0E-29 | 25% | 1.0E-66 | 29% |
| 2 | scaffold_46931_2 | 4.0E-101 | 33% | 1.0E-110 | 34% | 1.0E-46 | 27% | 2.0E-103 | 33% | 3.0E-39 | 27% | 4.0E-58 | 28% |
| 3 | scaffold_28587_2 | 0.0E+00 | 46% | 0.0E+00 | 45% | 3.0E-35 | 23% | 0.0E+00 | 51% | 1.0E-33 | 23% | 4.0E-51 | 28% |
| 4 | scaffold_63763_5 | 2.0E-90 | 31% | 2.0E-86 | 30% | 5.0E-50 | 26% | 1.0E-109 | 33% | 1.0E-29 | 25% | 1.0E-66 | 29% |
| 5 | scaffold_6500_7 | 1.0E-130 | 36% | 1.0E-133 | 36% | 9.0E-44 | 27% | 5.0E-160 | 37% | 9.0E-29 | 24% | 2.0E-64 | 27% |
| 6 | scaffold_6500_2 | 2.0E-99 | 33% | 3.0E-102 | 33% | 4.0E-45 | 28% | 5.0E-134 | 35% | 4.0E-26 | 23% | 7.0E-43 | 27% |
| 7 | scaffold_10015_1 | 4.0E-112 | 35% | 6.0E-108 | 34% | 4.0E-37 | 29% | 1.0E-125 | 36% | 1.0E-19 | 22% | 2.0E-38 | 27% |
| 8 | scaffold_25652_75 | 2.0E-91 | 33% | 4.0E-90 | 32% | 1.0E-39 | 25% | 1.0E-97 | 31% | 1.0E-28 | 23% | 1.0E-58 | 27% |
| 9 | scaffold_6500_12 | 2.0E-86 | 31% | 2.0E-86 | 32% | 5.0E-38 | 25% | 2.0E-90 | 30% | 7.0E-26 | 23% | 1.0E-64 | 29% |
| 10 | scaffold_15204_48 | 8.0E-81 | 31% | 2.0E-84 | 31% | 2.0E-35 | 25% | 2.0E-82 | 30% | 3.0E-32 | 24% | 2.0E-51 | 27% |
| 11 | scaffold_29233_1 | 6.0E-82 | 33% | 3.0E-78 | 32% | 5.0E-31 | 26% | 5.0E-76 | 31% | 3.0E-23 | 25% | 4.0E-51 | 28% |
| 12 | scaffold_14680_83 | 9.0E-80 | 28% | 4.0E-85 | 29% | 2.0E-35 | 25% | 2.0E-79 | 29% | 5.0E-31 | 24% | 2.0E-61 | 28% |
| 13 | scaffold_8379_70 | 3.0E-97 | 33% | 2.0E-96 | 32% | 3.0E-34 | 26% | 2.0E-106 | 32% | 1.0E-29 | 25% | 1.0E-69 | 29% |
| 14 | scaffold_8379_74 | 2.0E-79 | 28% | 2.0E-85 | 29% | 9.0E-42 | 26% | 3.0E-92 | 29% | 7.0E-35 | 27% | 1.0E-57 | 27% |
| 15 | scaffold_8379_64 | 4.0E-86 | 32% | 4.0E-85 | 31% | 2.0E-33 | 27% | 9.0E-98 | 30% | 2.0E-25 | 24% | 2.0E-56 | 26% |
| *NCBI BLAST was also performed on protein sequences against aerobic acetylenotroph *Mycobacterium lacticola*, but returned no matches. | | | | | | | | | |  |  |  |  |
